# Computational Prediction of Polycomb-Associated Long Non-Coding RNAs

**DOI:** 10.1371/journal.pone.0044878

**Published:** 2012-09-13

**Authors:** Galina V. Glazko, Boris L. Zybailov, Igor B. Rogozin

**Affiliations:** 1 Division of Biomedical Informatics, University of Arkansas for Medical Sciences, Little Rock, Arkansas, United States of America; 2 Department of Biochemistry and Molecular Biology, University of Arkansas for Medical Sciences, Little Rock, Arkansas, United States of America; 3 National Center for Biotechnology Information, National Library of Medicine, National Institutes of Health, Bethesda, Maryland, United States of America; University of Turin, Italy

## Abstract

Among thousands of long non-coding RNAs (lncRNAs) only a small subset is functionally characterized and the functional annotation of lncRNAs on the genomic scale remains inadequate. In this study we computationally characterized two functionally different parts of human lncRNAs transcriptome based on their ability to bind the polycomb repressive complex, PRC2. This classification is enabled by the fact that while all lncRNAs constitute a diverse set of sequences, the classes of PRC2-binding and PRC2 non-binding lncRNAs possess characteristic combinations of sequence-structure patterns and, therefore, can be separated within the feature space. Based on the specific combination of features, we built several machine-learning classifiers and identified the SVM-based classifier as the best performing. We further showed that the SVM-based classifier is able to generalize on the independent data sets. We observed that this classifier, trained on the human lncRNAs, can predict up to 59.4% of PRC2-binding lncRNAs in mice. This suggests that, despite the low degree of sequence conservation, many lncRNAs play functionally conserved biological roles.

## Introduction

Functional annotation of the noncoding part of transcriptome (70–90% of transcribed matter [1], [2], [3]) remains inadequate. Noncoding RNAs (ncRNAs) is a broad class of transcripts, consisting of well known transcripts with structural (rRNAs, tRNAs, snRNAs, snoRNAs, etc.) and regulatory (miRMAs, piRNAs, etc.) roles, and transcripts whose functions remain largely unknown [3], [4], [5]. The latter includes sense/antisense transcripts, ranging in length from 200 bp to 100 kb. Collectively they are called long non-coding RNAs (lncRNAs) [4] and sometimes referred to as genomic ‘dark matter’ [6]. A once-popular view that lncRNAs are by-products of the background transcription, i.e. “simply the noise emitted by a busy machine” [7] was related to their low abundance and poor evolutionary conservation. However, some large-scale evolutionary properties of the bulk of lncRNAs [8] and the existence of approximately two hundreds of experimentally characterized lncRNAs [9], [10] suggest that many of them have well-defined biological function [5], [6]. The first large-scale computational annotation of lncRNAs [11] has been based on the ‘guilt-by-association’ principle [12]. In this study lncRNAs were functionally characterized from their coexpression with - as well as their genomic adjacency to - protein-coding genes [11].

Initially, lncRNAs were found by ‘tiling arrays’, which are overall similar to microarrays but differ from them in the nature of probes, allowing for coverage of the entire genome at high resolution [2], [13], [14], [15], [16]. Many of these transcripts appeared at very low detection levels, close to the detection limit of qPCR and Nothern blots [17]. Another technology was employed by the FANTOM consortium which revealed over 3,500 mouse non-coding transcripts, also at low abundance and poor sequence conservation [1], [18]. Recently, Guttman et al. [19] identified approximately 1,600 lncRNAs located in intergenic regions (long intergenic noncoding RNAs, lincRNAs), enriched in a certain chromatin signature, which is characteristic of active transcription (K4–K36 domains) in four mouse cell types. Surprisingly, only a small fraction (∼11%) of the Guttman et al. [19] set matched the FANTOM lncRNA catalogue [20]. Finally, transcriptome sequencing (RNA-Seq) – the technology that has for a large part supplanted microarrays because it does not suffer from cross-hybridization [21] and is able to accurately detect expression at the lower end of the dynamic range of the transcriptome [22] – was applied to study the ‘dark matter’ transcriptome [23]. It has been shown that many of the transcripts identified using tiling arrays were, indeed, false positives. Nevertheless, the presence of thousands of low-abundance, unannotated transcripts was confirmed (22% of reads in human and 51% in mouse) [23]. Thus, the existence of lncRNA transcripts, albeit at low abundance, has been repeatedly confirmed at different levels of technical resolution. Yet, the existence by itself does not answer the question of whether these lncRNAs have functions, or whether most of them are mere byproducts of active transcription of the protein-coding genes.

Among the thousands of lncRNAs, ca. forty mammalian lncRNAs are functionally characterized in detail [9]. Recently, Lee [24], [25] suggested that the low abundance *Xist* lncRNA, which is involved in X chromosome inactivation, exemplifies a regulatory model for other lncRNAs. Lee’s “guides and tethers” hypothesis states that lncRNAs may contain binding sites for chromatin modifiers and may serve as “tethers” that recruit chromatin modifiers to their own genomic address during *in cis* regulation, while others may guide these complexes to other locations *in trans*. This hypothesis suggests lncRNAs as potential regulators of spatial and temporal expression during development, because the RNA transcription itself occurs in a developmentally-specific manner [24], [25]. Recently discovered the low-copy lncRNA HOTTIP, which is transcribed from the 5′ of HOXA locus [26], regulates *in cis* the expression of several 5′ HOXA genes. Another HOX-associated lncRNA HOTAIR, transcribed from the HOXC locus, targets PRC2 and is necessary for HOXD locus silencing *in trans* [27]. It also may serve as a scaffold for targeting of chromatin modifier complexes PRC2 and LSD1 to hundreds of genes across the genome for silencing [28]. Several other lncRNAs were recently functionally characterized (see [29] for a review), and their various functionalities can be summarized as large-scale, tissue-specific, and developmental regulation of gene expression.

Functional annotation of lncRNAs on a genomic scale has been elusive for a long time [9]. Recently, however, experimental studies have demonstrated that more than 20% of lncRNAs in human and many lncRNAs in mouse are bound by PRC2 [30], [31]. Polycomb proteins are conserved in flies and mammals and PRC2 complex represses the transcription of specific genes via trimethylation of H3K27 (see [32] for a review). While in *D.melanogaster* Polycomb proteins are recruited to their target genes via GA-rich DNA-sequence elements, called Polycomb Response Elements (PREs), PREs in mammals were not identified. The observations that thousands of lncRNAs are bound by PRC2 in human as well as in mouse cell lines [30], [31], and recent data on numerous lncRNAs occupancy sites [33] indicate the possibility of the functional classification of lncRNAs on a genome-wide scale into two classes: lncRNAs that function similarly to PREs and bind PRC2 complex in mammals and the rest of lncRNAs.

The functional similarity between PREs and PRC2-binding lncRNAs triggers the question whether the PRC2-binding lncRNAs constitute a set of closely similar sequences, as PREs do. The answer to this question is negative: known PRC2-binding lncRNAs are too diverse to be alignable at the sequence level (see Materials and Methods section for human and mouse local and global alignment scores between PRC2-binding lncRNAs) and do not share obvious structural similarities. The question, however, can be stated more generally: Do PRC2-binding lncRNAs constitute a family where members share similar sequence-structure patterns, in the same way as do the members of the families in the RNA families (Rfam) database [34]? If they do, then, based on the sequence-structure features, the PRC2-binding lncRNAs can be distinguished from the rest of lncRNAs. To the best of our knowledge this question has not been answered yet.

Supervised learning has been used for a variety of complex sequence-related problems in molecular biology for decades (e.g. [35]). Whether or not the two lncRNAs classes can be distinguished from one another can be reliably answered in the supervised learning framework. We consider three relatively modern machine learning techniques, namely support vector machine (SVM) (see [36] for a review), Shrinkage Discriminant Analysis (SDA) (introduced as a classification approach in [37]), Random Forest (RF) [38], and also one classical approach, Logistic Regression (LR) [39]. Using the catalogue of human PRC2-binding and PRC2 non-binding lncRNAs [30], we first identify sequence-structure features that are significantly different between the two lncRNAs classes. Second, we construct four different classifiers (SVM, SDA, RF and LR), test their performance via leaving-one-out cross-validation (LOOCV), and identify SVM-based classifier as the one with the highest performance and the lowest misclassification rate. Third, we demonstrate that the SVM-based classifier correctly predicts several known PRC2-binding lncRNAs and also can predict up to 59.4% of PRC2-biding lncRNAs in murin embryonic stem cells.

In this study we demonstrate the existence of the two functionally different lncRNAs classes computationally. We believe that these two classes occupy only a small area inside the lncRNA functional landscape and more detailed functional classification of lncRNAs will become available as experimental data continues to accumulate.

## Results

Molecular mechanisms of how lncRNAs regulate transcripts can be divided into those mediated through: (1) interactions with PRC2 and other chromatin modifiers; (2) interactions with binding sites for general transcription factors (TFs) and interactions with proteins recognizing dsRNAs; (3) base-paring of a candidate gene and the complementary antisense [3], [40], [41], [42]. Below we describe these mechanisms in more detail.

### PRC2 Chromatin Modifier

About 20% of lncRNAs expressed in different cell types are bound by the Polycomb chromatin repressive complex PRC2 [30]. When additional chromatin modifiers are included, the proportion of bound lncRNAs becomes 38% [30]. The exact molecular mechanism, beyond the shown physical interaction, is unknown. However, there are several examples of the role of the secondary structure in binding. For example, RepA Xist (the internal noncoding transcript from the Xist locus) that folds into a conserved stem-loop structure [43], binds PRC2 directly and is sufficient to recruit PRC2 *in vivo* without Xist [44], [45]. The analysis of lncRNA HOTAIR, which also interacts with PRC2 [28], has demonstrated that secondary structures of exons 1 and 6 are conserved among five mammalian species [46]. Although RepA Xist has a different secondary structure than HOTAIR exons, one can hypothesize that PRC2 recognizes not a conservative secondary structure, but a set of structural patterns, for example, conserved stem-loops.

### dsRNA-binding Site

Recently it was discovered that certain lncRNAs that contain a single Alu element can interact with an Alu element in mRNA 3′UTR forming a binding site for proteins that recognize dsRNA [42].

### General TF -binding Sites

Some lncRNAs tend to be derived specifically from enhancer and promoter regions and may contain TF binding sites [4]. Some may even regulate transcription of the corresponding transcription factors via feedback loop, as was shown for four evolutionary conserved lncRNAs encoded at loci with Nanog and Oct4 binding sites [47].

### Sense-antisence Interaction

Many lncRNAs are transcribed antisense to the corresponding genes. They regulate the cognate gene expression via many different molecular mechanisms (from changing chromatin conformation to alternative IRS site) [3], [40].

The aforementioned mechanisms of how lncRNAs regulate gene expression can be viewed as an unknown combination of sequence-structure features, such as: (1) secondary structure patterns; (2) transcription factor binding sites; (3) short oligonucleotides describing sequence content and; (4) the repeat structure of the region (sequence complexity).

### Features Evaluation

We hypothesize that certain combinations of sequence-structure features can reliably distinguish between PRC2-binding and the rest of lncRNAs. The set of all patterns we considered is listed in the Materials and Methods section. RNA sequence-structure patterns (RSSPs) employed here were 397 RSSPs describing 42 highly structured families from Rfam10 database [48]. Motif binding sites were extracted as 1314 Position-Weight Matrices (PWMs) from Jaspar Transcription Factor Binding Profile database [49]. As short oligonucleotides we considered all *k*-words of length *k* = 2, 3, 4, 5, 6, 7, 8. The sequence complexity was measured as an approximation of Kolmogorov Complexity (KC) using the Lepel-Ziv data compression algorithm. KC [50] is a characteristic of sequence ‘randomness’ and is inversely related to the number of repetitive elements.

To prepare sets of positive and negative examples for classifiers training we carefully filtered PRC2-binding and PRC2 non-binding lncRNAs presented by Khalil et al. [30] (see Material and Method for detail). We obtained 314 and 454 PRC2-binding and PRC2 non-binding lncRNAs, respectively, followed by a random selection of 314 sequences from the set of negative examples to equalize the sample sizes.

First, we identified sequence-structure features, which are significantly different between the two lncRNAs classes. As a feature selection criterion, a two-sample *t*-test at two different significance levels (liberal, p<0.05 and conservative, p<0.01) was employed. The two significance levels (in addition to cross-validation) were selected to assess the overfitting problem, which could happen if too many features are selected at a liberal significance level (see below).

The number of short oligonucleotides (*k*-words), significantly different between the two lncRNAs classes growth with the lengths of k is shown in Table 1. Dinucleotide and trinucleotide frequencies were not significantly different, while there were already 1104 *k*-words, significantly different for *k* = 8. To characterize these oligonucleotides qualitatively we constructed consensus sequences for all *k*-words (*k* = 4,…,8), significantly enriched in PRC2-binding and PRC2 non-binding lncRNAs separately. As the Table 1 shows, for PRC2-binding lncRNAs and different *k* the consensus sequences are represented by consistent AT-rich signature. Interestingly, there is no consistent signature for lncRNAs that do not bind PRC2 (Table 1). This observation indicates that, indeed, sequence-structure patterns are different between the two classes of lncRNAs and lncRNAs that do not bind PRC2 constitute more diverse set of sequences. The latter observation is supported by values of KC for the two classes: the complexity of PRC2-binding lncRNAs is significantly lower (p<0.01) than the complexity of PRC2 non-binding lncRNAs. We selected the length *k* = 6 (196 oligonucleotides) as a descriptive length for oligonucleotide features to be included in the prediction rule. This value of *k* is a reasonable compromise between the number of features and the information content of the signal. At the more conservative significance level 29 oligonucleotides were significantly different between the two classes (*k* = 6).

**Table 1:** Consensus motifs enriched in PRC2-binding and PRC2 non-binding lncRNAs.

Length	Number of motifs	Enriched in PRC2^+*a)*^	Enriched in PRC2^−*b)*^
k = 4	15	WWHH c)	SBSC
k = 5	47	TYKWW	SSYCV
k = 6	196	WWWRW	SKSCSM
k = 7	568	WWWWWW	SYSMSMS
k = 8	1104	WWWWWWWW	BHMRVMAD

a) PRC^+^: PRC2-binding lncRNAs.

b) PRC^−^: PRC2 non-binding lncRNAs.

c) IUPAC nucleotide code: http://www.bioinformatics.org/sms/iupac.html.

There were 68 motifs represented as PWMs, which were significantly different between the two lncRNAs classes at the 0.05 level of significance. These motifs include various binding sites for transcription factors, mostly represented by helix–turn–helix and zinc finger classes. We were not able to discern a straightforward trend for the transcription factor binding sites overrepresented in the two different classes of lncRNAs. This observation is most likely related to: (1) diversity of lncRNAs binding proteins in the both classes and (2) low sequence specificity of the transcription factors. At the conservative significance level there were only 5 sites, different between the two classes.

There were no RSSPs significantly different between the two classes at different significance levels. This result might be related to the choice of RSSPs (see Material and Methods section): only highly structured RFAM10 families (all families with a consensus secondary structure containing at least 5 stem-loop substructures) were included. This criterion may be too restrictive, and if less stringent RSSPs are used the results can be different. We defer the detailed answer to this question for the future studies.

Finally, the two sets of features consisting of {6-words, KC, motifs} at 0.05 (265 features) and 0.01 (35 features) significance levels were formed. The extent of overfitting was evaluated from classifier performance (see below).

### Evaluation of Classifiers

We evaluated the performance of four different classifiers: support vector machine with linear kernel (SVM) [36], Shrinkage Discriminant Analysis (SDA) [37], Random Forest (RF) [38], and Logistic Regression (LR) [39]. However, all of them have a nice property in common: they behave better than the classical approaches (e.g. Fisher LDA) for high-dimensional data sets, containing more variables than observations. Their performance was evaluated with five different performance measures, namely specificity, sensitivity, misclassification rate, accuracy, and an empirical area under the curve (AUC). To avoid overfitting, all performance measures were estimated using LOOCV, where a single observation from the original sample is considered as the testing sample, and the rest is used as the training samples; the process is repeated for all observations. Because the feature selection step is not included in the cross-validation, the error rate is not expected to be overoptimistic [51]. Hyperparameter tuning for SVM was performed as the nested cross-validation.

**Figure 1:**
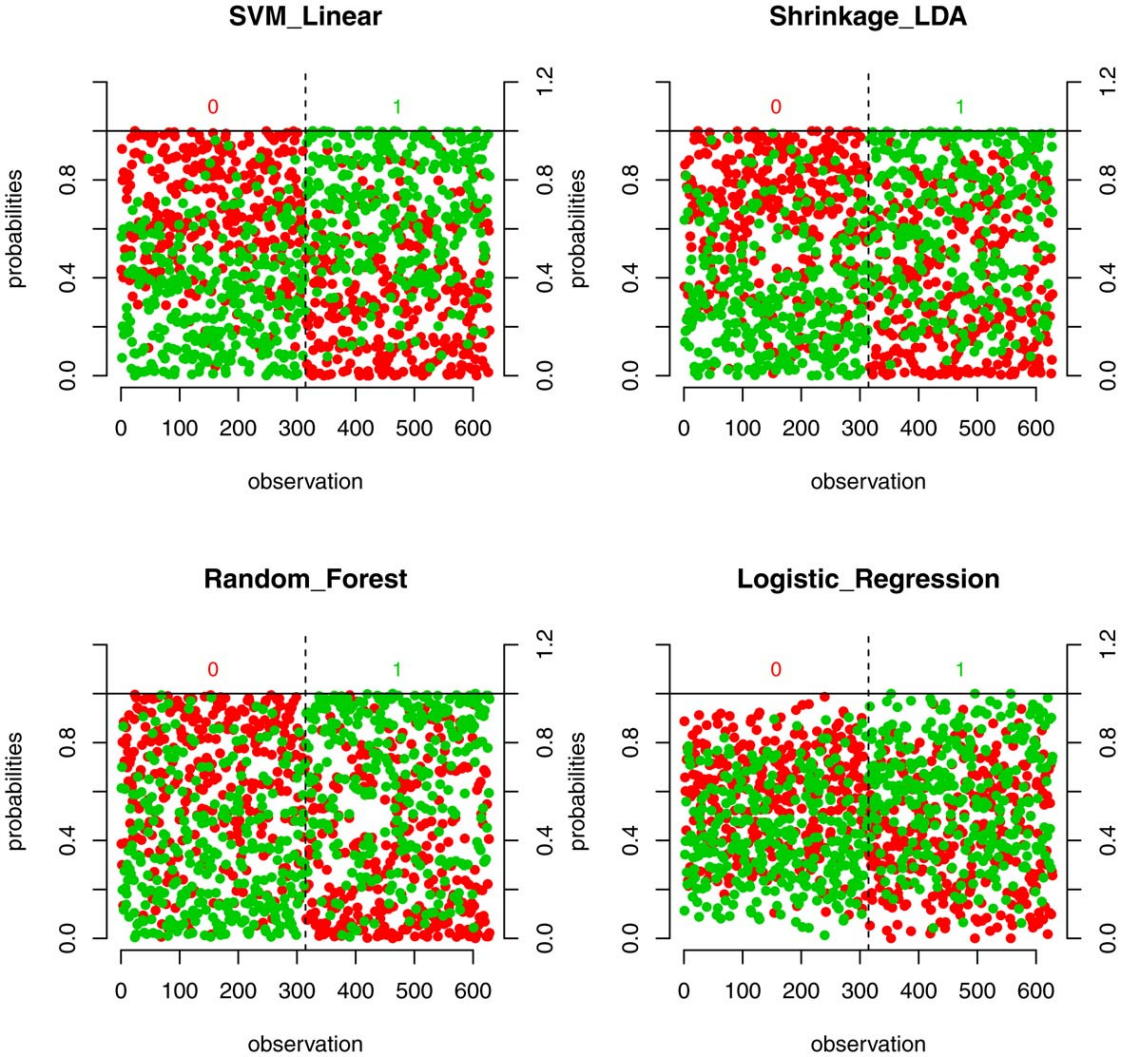
Visualization of the classification performance for four classifiers and the set of features, selected at the 0.05 significance level. The observations along X axis are reordered according to their true class labels. For each observation red and green dots represent the estimated probabilities to belong to class 0 and 1 respectively. Dotted line separates observations from class 0 and class 1. As it is evident from the plot, the probability of observation to belong to a specific class is in agreement with its class label.

**Figure 2:**
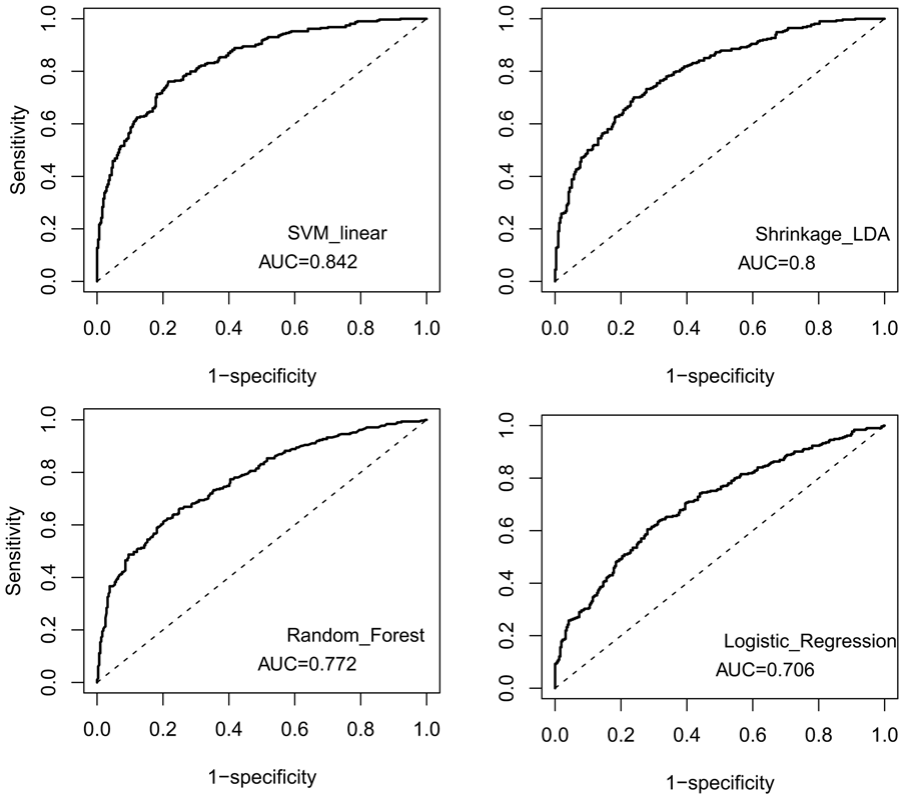
**ROC curves for four different classifiers and the set of features selected at the 0.05 significance level.**

We tested the performance of different classifiers given the set of features selected at the 0.05 significance level. The visualization of the performance based on the probability to belong to a particular class is shown in the Figure 1. It can be seen that SVM and SDA yield slightly better separation of the two classes than RF and LR. Indeed, the areas under the curves computed from receiver operating characteristic curves (ROCs) for SVM and SDA are larger than for RF and LR (Figure 2). The full evaluation of the performance is presented in Table 2. The SVM with linear kernel has higher specificity and sensitivity and lower misclassification rate, as compared to other classifiers. For the set of 35 features, the class separation for all classifiers was worse, but still sufficient for classification (SVM accuracy: 0.697). This indicates that the possibility of overfitting due to the large number of parameters is not an issue. The full evaluation of performance of the classifiers is presented in Table 3. Interestingly, the performance of SVM and SDA is always better than those of Random Forest and Logistic Regression classifiers (Tables 2, 3); however the performance of SVM classifier is always better than that of SDA. We also studied the performances of different classifiers with normalized and non-normalized data. The performance of SDA classifier was significantly worse and the performance of LR classifier was significantly better for standardized data. We therefore normalized the data in the case of LR classifier only.

**Table 2:** Classifiers performances (0.05 significance level).

Classifier	Specificity	Sensitivity	Misclassification	Accuracy
SVM linear	0.787	0.745	0.234	0.766
Shrincage LDA	0.771	0.682	0.274	0.726
Random Forest	0.704	0.682	0.307	0.693
LogisticRegression	0.688	0.631	0.341	0.659

Based on the evaluation results we build SVM-based classifier with the linear kernel given the set of 265 features, in order to test the ability of classifier to generalize on the independent data sets.

### Predicting PRC2-binding lncRNAs using Independent Data Sets

#### Examples for human lncRNAs

Here we were interested to test the generalization property of the best performing SVM classifier with linear kernel. There are not so many examples of specific PRC2-binding lncRNAs for human. Therefore we selected only four, yet well documented examples of PRC2-biding human lncRNAs: three fragments of HOTAIR (1–300, 1–1500 PRC2-binding, and 1500–2158 PRC2 non-binding fragments), and also RepA Xist. As a different control set we downloaded the genome-wide coordinates of PRC2-binding lncRNAs in mouse embryonic stem cells. There were 215 PRC2-binding mouse lncRNAs (Supplementary Table S4 in [31]). After filtering (see Materials and Methods) 106 sequences were left.

**Table 3:** Classifiers performances (0.01 significance level).

Classifier	Specificity	Sensitivity	Misclassification	Accuracy
SVM linear	0.688	0.707	0.303	0.697
Shrincage LDA	0.707	0.688	0.303	0.693
Random Forest	0.669	0.659	0.336	0.632
LogisticRegression	0.640	0.694	0.333	0.667

LncRNA HOTAIR has two different binding activities: with a series of HOTAIR deletion mutants it was shown that PRC2 binds nucleotides 1–300 of HOTAIR, while the fragment 1500 to 2146 binds LSD1 complex, mediating enzymatic demethylation [28]. The authors suggested that HOTAIR bridges these two complexes together, acting as a modular scaffold [28]. Interestingly, SVM-based classifier was able to correctly identify the regions of 1–300 and 1500–2146 as PRC2-binding and PRC2 non-binding lncRNAs, respectively. RepA Xist is well documented example of PRC2-binding lncRNA [44], [45]. In our experiment the classifier also correctly identified RepA Xist as PRC2-binding (Table 4). Because RepA Xist in mouse and human are identical, RepA mouse was also correctly identified as PRC2-binding.

#### Examples for mouse lncRNAs

It is well known that the evolutionary sequence conservation of lncRNAs is poor ([6] and see Materials and Methods section). Surprisingly, for 106 mouse PRC2-binding lncRNAs the SVM classifier correctly predicted 63 as PRC2-binding. This observation testifies that the built classifier generalizes well on independent data sets and that PRC2-binding lncRNAs are well conserved not at the sequence level, but at the level of sequence-structure features.

HOTAIR is poorly conserved in placental mammals and does not exists in platypus or the other vertebrates [46]. In addition, sequence-structure conservation is present for first and sixth exons of HOTAIR only [46]. Recent study of mouse HOTAIR (mHOTAIR) has shown that the complete deletion of HoxC cluster (including mHOTAIR) in mouse embryos had virtually no effect on HoxD genes [52], though human HOTAIR was shown to act *in trans* and regulate the expression of HoxD genes [28]. Should the unexpected difference in the functional role of HOTAIR in human and mouse be explained by high sequence-structure divergence resulted in functional divergence, as suggested [52]? This question of course cannot be answered *in silico*, but it is still useful to test, even *in silico*, whether mHOTAR can be classified as PRC2-binding.

**Table 4:** SVM performance on independent data sets.

LncRNA	PRC2-binding	PRC2 non-binding
HOTAIR 1–300	1	0
HOTAIR 1–1500	1	0
HOTAIR 1500–2146	0	1
repA XIST (human, mouse)	1	0
106 mouse PRC2-binding	63	43
mHOTAIR 1–500	1	0
mHOTAIR 500–2006	0	1

**Figure 3:**
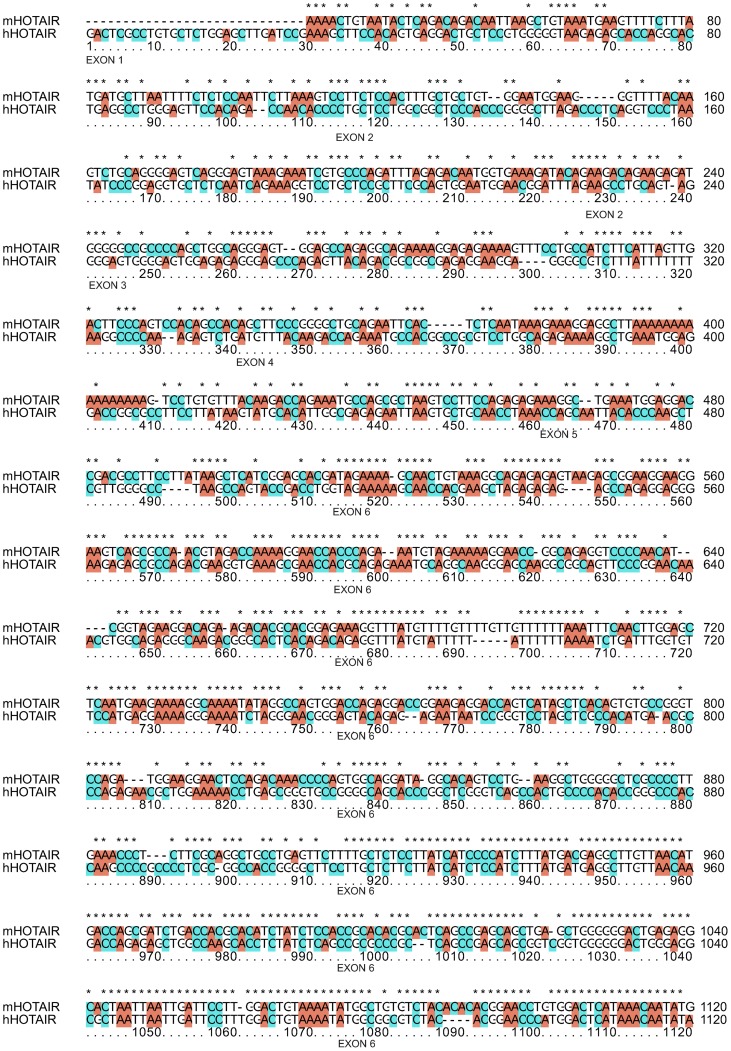
A fragment of mouse (mHOTAIR) and human (hHOTAIR) HOTAIR lncRNA alignment (positions 1–1120 in human lncRNA are shown). Exons coordinates are from NC00012.

As it was already observed by others, the alignment of human and mouse HOTAIR is poor. Only scarce fragments of sequence conservation for human exons 1–5 exist in mHOTAIR (Figure 3). However, starting from exon 6, the conservation becomes more pronounced (Figure 3). We tested the first 500 and the last 500 bp of mHOTAIR. The SVM classifier predicted the most poorly conserved (exons 1–5 in human, see Figure 3) the first 500 bp of mHOTAIR as PRC2-binding, and the last 500 bp of mHOTAIR as PRC2 non-binding lncRNA (Table 4), as in the case of human HOTAIR. This observation suggests that it is not the sequence *per se*, but the combination of sequence-structure features that is important for PRC2 binding. It would be interesting to test mHOTAIR in direct experiment for PRC2-binding. The fact, that the expression of HoxD genes is seemingly not altered by the deletion of HoxC cluster [52] does not proof with necessity that mHOTAIR does not bind PRC2.

## Discussion

The small-scale functional annotation of lncRNAs is an area of active research [4], [5], [9], but is almost non-existing at the genomic scale because of the lack of experimental data [9], [11]. However, the interaction of lncRNAs and Polycomb repressive complex (PRC2) is well documented and the experimental approaches capturing PRC2-associated transcriptome are rapidly developing [30], [31]. In this study we demonstrate that a set of lncRNAs can be confidently split into two different classes: PRC2-binding and PRC2 non-binding.

This classification is enabled by the fact that while all lncRNAs constitute a diverse set of sequences, the classes of PRC2-binding and PRC2 non-binding lncRNAs possess characteristic combinations of sequence-structure patterns and, therefore, can be separated within the features space. Based on this observation, using a combination of sequence-structure patterns constructed from transcription factor binding sites, Kolmogorov complexity, and oligonucleotide frequencies, we built several machine-learning classifiers and identified SVM-based classifier as the best performing. The evaluation of SVM classifier performance on the training data set is promising: the accuracy (0.766) is reasonably high and the accuracy on the smaller set of features (0.697) is also acceptable. This indicates that the possibility of overfitting due to the large number of parameters is negligible.

The SVM-based classifier generalizes well on independent data sets. Different fragments of human HOTAIR (first and last exons) were correctly predicted to possess PRC2-binding and PRC2 non-binding activities, respectively [28]. Well-known PRC2-binding lncRNA repA Xist was also correctly classified. The surprising observation that we were able to correctly predict 59.4% of PRC2-binding mouse lncRNAs has an important biological implication that many lncRNAs are evolutionary conserved, but only at the level of sequence-structure patterns. This observation was further strengthened by the case of mouse HOTAIR lncRNA. Despite poor conservation of human HOTAIR exons 1–5 in mouse, the first 500 bp of mHOTAIR were identified as PRC2-binding, and the last 500 as PRC2 non-binding, similar to the case of human HOTAIR. Although without experimental confirmation it cannot be stated that mHOTAIR binds PRC2 similarly to human HOTAIR, this fact may at least serve as a demonstration that despite sequence differences, the similarity between human and mouse HOTAIR lncRNAs is traceable, albeit on the other level.

The drawback of our classifier performance study on the independent data sets is the absence of negative examples. That is, while we see that the SVM-based classifier has high sensitivity its specificity is still unknown. This is because a reasonable set of negative examples is difficult to find: rarely in an experiment would one try to proof that a given lncRNA does not bind PRC2. In addition, by construction the classifier can be used for lncRNAs only.

The existence of distinguishable lncRNAs classes may also indicate that not all lncRNAs are evolving fast. While, as in the case of mouse PRC2-binding lncRNAs, the evolutionary rate can be high because it may not imply the loss of function, the class of PRC2 non-binding lncRNAs may have other functional requirements and evolve more slowly. Obviously, lncRNAs regulating expression of genes in antisense should evolve at the rate, similar to overlapped regions of their cognate genes.

With our ability to classify and functionally annotate lncRNAs the view that lncRNAs are by-products of the background transcription is refuted. Further large-scale experiments will allow classifying lncRNAs into more functionally homogeneous classes and the ability to computationally predict whether a given lncRNAs is functioning *in cis* or *in trans* for a subset of *in cis* lncRNAs will uncover their targets. In conclusion we note that computational approaches for functional characterization of lncRNAs may soon become as important as computational approaches for predicting protein coding genes became two decades ago.

## Materials and Methods

### Data Sources

#### Training samples

We selected as PRC2-associated lncRNAs the intersection of coordinates of lncRNAs bound to polycomb repressive complex 2 in the 3 cell types (table 3 in Dataset S1, [30]) and coordinates of lncRNA exons defined by Nimblegen tiling microarrays (table 2 in Dataset S1, [30]). We note that although positive and negative examples of lncRNAs were taken from the intergenic regions (called ‘lincRNAs’ in the original study), we use the term lncRNAs everywhere in our manuscript for the sake of generality. As for lncRNAs, that do not associate with PRC2 we selected the rest of coordinates of lncRNA exons. That is, in the positive set of examples we included sequences given by the intersection of coordinates from tables 3 and 2, and in the negative set of examples we included sequences with coordinates from table 2, without coordinates from the intersection of tables 2 and 3. Both data sets were filtered: only lncRNAs that were also found in human ESTs were retained for comparison; only sequences more than 100 bp were retained, resulting in 314 and 454 sequences. To increase the sample size, instead of the cutoff of 200 nucleotides, commonly used to define lncRNAs, we used the 100 nucleotides cutoff. It should be noted that the most up-to-date definition of lncRNAs as “RNA molecules that may function as either primary or spliced transcripts and do not fit into known classes of small RNAs” [53] does not include any cutoff based on the sequence length. To equalize the sample sizes 314 sequences from the set of negative examples were selected at random. Both sets are available in the Data File S1.

#### Sequence characteristics of the training samples

The ranges of sequence lengths in the sets of positive and negative examples were 101–7078 bp and 100–3235 bp, respectively (Figure S1). To estimate the sequence similarities in training samples we implemented the global (Needleman-Wunsch) and the local (Smith-Waterman) pairwise alignments for every set (49141 local and 49141 global alignments per set). Nucleotide matches and mismatches were scored 1 and -3, respectively. For the global alignment affine gap penalty score -10 was used. There were no identical sequences in both sets, and the sequence similarities were generally low (Figure S2, the scores distributions for global and local alignments in PRC2-binding - and PRC2 non-binding lncRNAs). The best global (local) alignment scores were -92 (172) and 0 (191) for the sets of positive and negative examples, respectively (Figure S2). Even for the best local alignment scores (172 and 191), the sequence identities (measured as the ratio of the number of the identical nucleotides in the alignment to the alignment length) were low (Figures S3 and S4).

#### Independent data sets

The genome-wide coordinates of PRC2-binding lncRNAs in mouse embryonic stem cells were downloaded from Supplementary Table 4 in [31]. There were 215 PRC2-binding mouse lncRNAs (Supplementary Table 4 in [31]). We selected only those lncRNAs that were found in mouse EST; only sequences more than 100 bp were left, resulting in 106 sequences set. The range of the sequence lengths was 101–5230 bp. Again, to estimate the sequence similarities in testing samples we implemented global (Needleman-Wunsch) and local (Smith-Waterman) pairwise alignments (5565 local and 5565 global alignments, the scoring matrix was the same as the one used for training samples). There were no identical sequences. The best global and local alignment scores were -157 and 132, respectively (Figure S5). For the best local alignment score 132 the sequence identity (measured as the ratio of the number of the identical nucleotides in the alignment to the alignment length) was low (Figure S6). We also estimated the sequence similarities between mouse PRC2-binding lncRNAs (106 sequences set) and the training set of human PRC2-binding and PRC2 non-binding lncRNAs. Again, there were no identical sequences. The best global (local) alignment scores were -127 (43) between human and mouse PRC2-binding lncRNAs and -130 (33) between mouse PRC2-binding - and human PRC2 non-binding lncRNAs (Figure S7). Local alignments for the best local alignment scores 33 and 43 are shown in Figures S8 and S9, respectively. It is clear that even between PRC2-biding lncRNAs from different species the sequence conservation is poor (Figure S9).

RepA Xist corresponds to position 292 to 713 in mouse (gi|37704378) and 350 to 770 in human (gi|340393) [54]. As mRNA for human HOTAIR we used gi|145688388. Mouse predicted HOTAIR RNA (Gm16258) corresponds to RefSeq AK035706 transcript [52].

### Sequence Features

#### RNA sequence-structure patterns (RSSPs)

We considered 397 RSSPs describing 42 highly structured families (all families with a consensus secondary structure containing at least 5 stem-loop substructures) from RFAM10 database, compiled by the authors of the Structator software [48].

#### Motifs and oligonucleotides

The frequencies of short oligonucleotides and motifs, represented as Position Weights Specific Matrices (1314 PWMs extracted from Jaspar database) were calculated using the Biostrings package in Bioconductor implemented in the R language [55]. For motifs, as the resulting frequency we took the average of frequencies over both strands.

#### Kolmogorov complexity

Formally, the absolute amount of information in a string is the size of the smallest program (*p*) of an optimal Turing Machine (*U*) that is needed for generating that string: *K(x)* = min{*|p|:U(p) = x*} and is called ‘Kolmogorov complexity’ (*K(x)* of a string *x*) [50]. In our case lncRNA sequence is such a string. To approximate Kolmogorov Complexity the well-studied Lempel-Ziv algorithm was employed [56].

### Performance Measures

The performance of all classifiers was estimated using leaving-one-out cross-validation (LOOCV), where each classifier is trained on (*N*-1) samples (where *N* is the sample size) and then tested on the one sample left, repeating this step *N* times. The performance was estimated as iterationwise average performance.

We estimated average
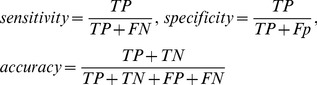
(1)and misclassification rate for every classifier. We also evaluated the performance of classifiers measuring receiver-operating characteristic curve (ROC) area under the curve (AUC).

### Implementation

When the number of observation (*n*) is less than the number of variables (*p*) popular approaches for classification (e.g. Linear Discriminant Analysis, LDA) do not perform well and other methods, introduced in the context of high-dimensional genomic data such as microarrays should be employed. Although in our case the number of variables has never exceeded the number of observations, an application of classical LDA resulted in poor performance (data not shown). We therefore selected relatively modern classifiers tuned for higher dimensionality. The most novel classifier considered here is the Shrinkage Discriminant Analysis, an extension of classical discriminant analysis, that overcomes the problem of not invertible covariance matrix when *n*<<*p* by using a covariance estimation procedure [37]. We also considered more established techniques, such as Support Vector Machine [36] and Random Forest (RF) [38], and as the most classical approach we employed Logistic Regression (LR) [39]. SVM classifier is known to be sensitive to the parameters, and its performance decreases significantly without tuning. We used here a linear kernel and the only tuning parameter was the cost (C). We considered a grid for the cost parameter and employed the nested cross-validation, resulting in the value of hyperparameter that gave the smallest misclassification rate (C = 0.1). All computations were performed in Bioconductor package CMA (‘**C**lassification for **M**icro**A**rrays) [57], implemented in the R language [55].

## Supporting Information

Data File S1 The sets of PRC2-binding and PRC2 non-binding lncRNAs (positive and negative examples) used in this study to train classifiers.
(TXT)

Figure S1 The distributions of sequence lengths for the sets of training samples.
(TIFF)

Figure S2 The distributions of local and global alignment scores for human PRC2-binding and PRC2 non-binding lncRNAs.
(TIFF)

Figure S3 The best local alignment between human PRC2-binding lncRNAs.
(TIFF)

Figure S4 The best local alignment between human PRC2 non-binding lncRNAs.
(TIFF)

Figure S5 The distributions of local and global alignment scores for mouse PRC2-binding lncRNAs.
(TIFF)

Figure S6 The best local alignment between mouse PRC2-binding lncRNAs.
(TIFF)

Figure S7 The distributions of local and global alignment scores between mouse PRC2-binding lncRNAs and 1) human PRC2 binding lncRNAs and 2) PRC2 non-binding lncRNAs.
(TIFF)

Figure S8 The best local alignment between mouse PRC2-binding and human PRC2 non-binding lncRNAs2.
(TIFF)

Figure S9 The best local alignment between mouse and human PRC2-binding lncRNAs.
(TIFF)
