# Supplementary figures and images for: Computational Prediction of Polycomb-Associated Long Non-Coding RNAs
Source: PLoS One. 2012 Sep 13;7(9):e44878. doi: 10.1371/journal.pone.0044878 (PMC3441527; doi:10.1371/journal.pone.0044878)

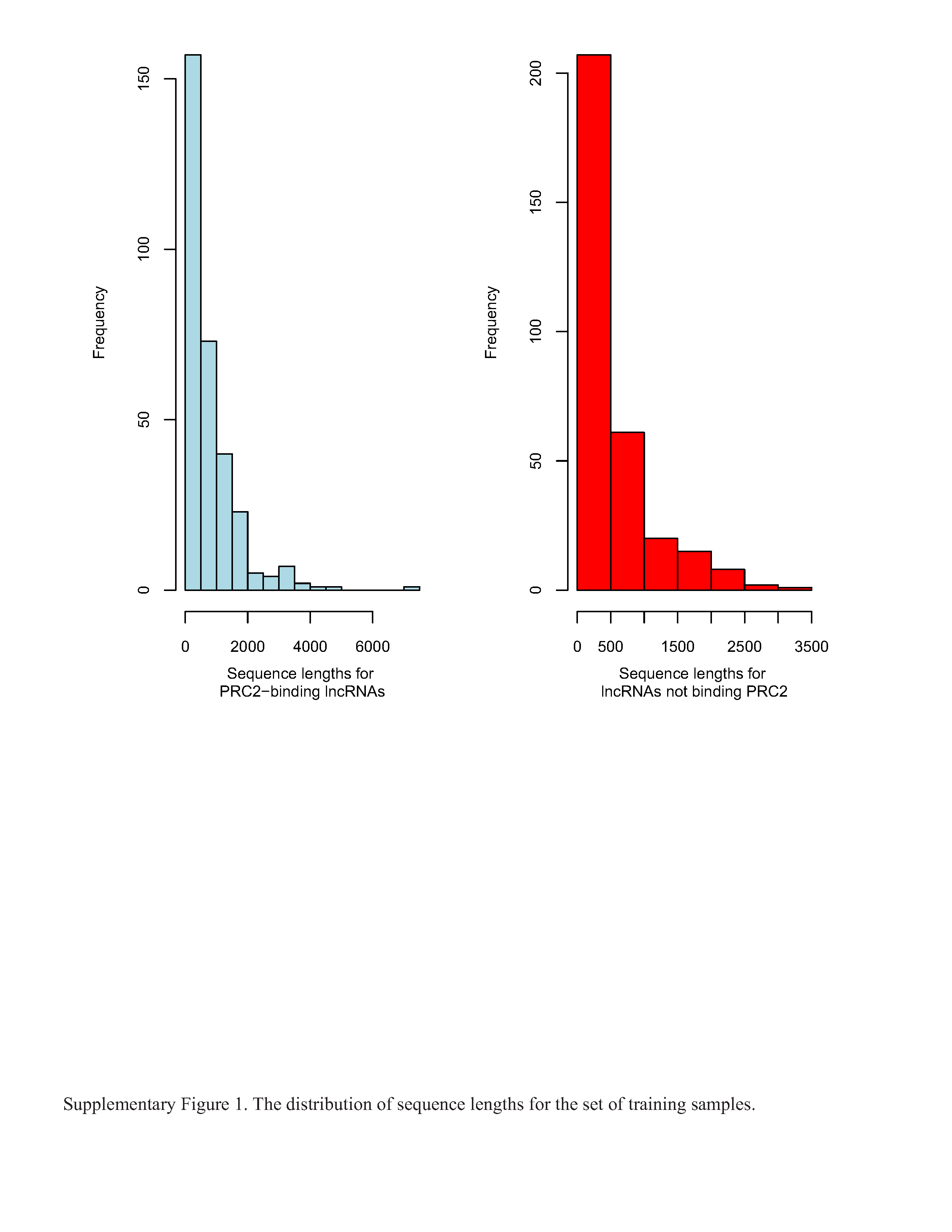

Supplement: Figure S1 — The distributions of sequence lengths for the sets of training samples. (TIFF) [file pone.0044878.s002.tiff]

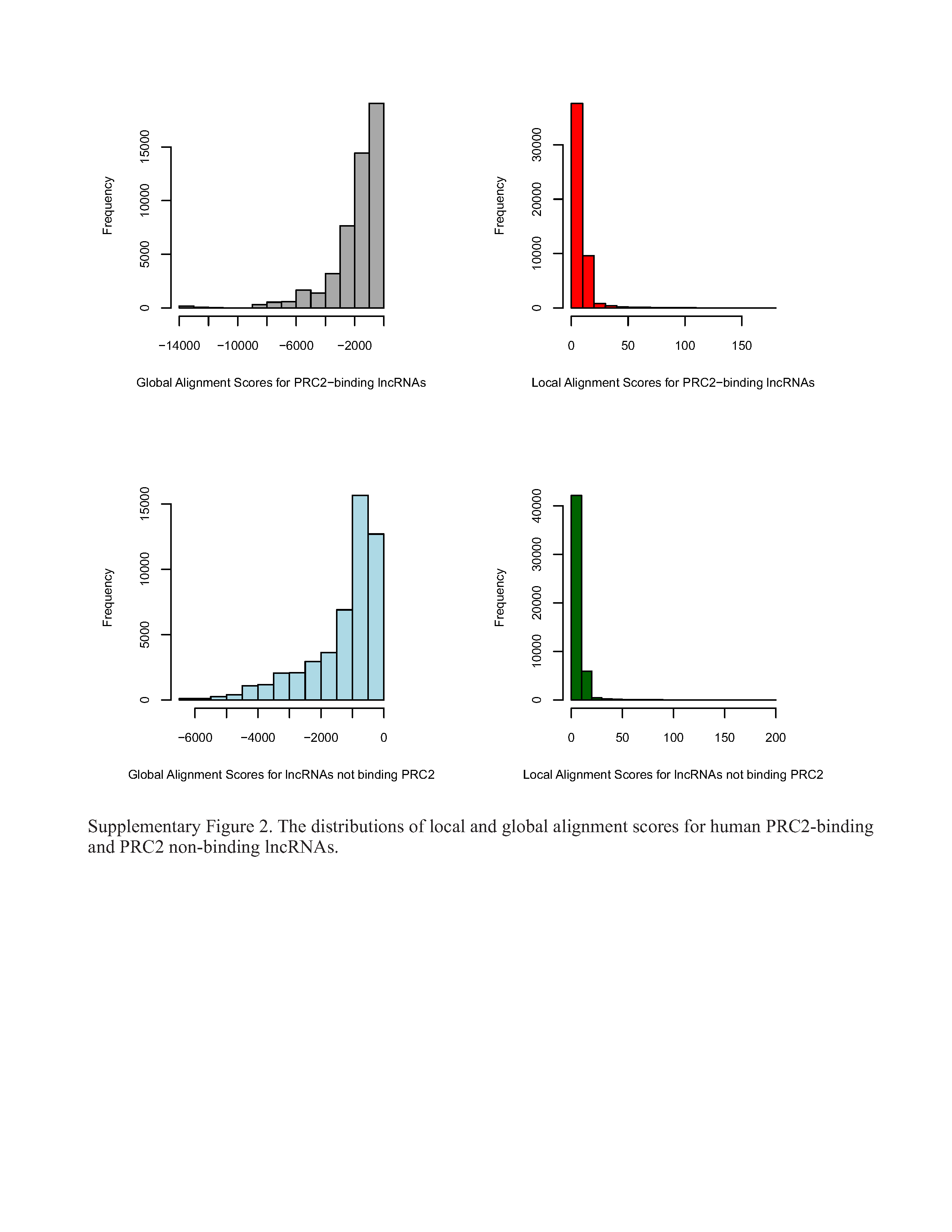

Supplement: Figure S2 — The distributions of local and global alignment scores for human PRC2-binding and PRC2 non-binding lncRNAs. (TIFF) [file pone.0044878.s003.tiff]

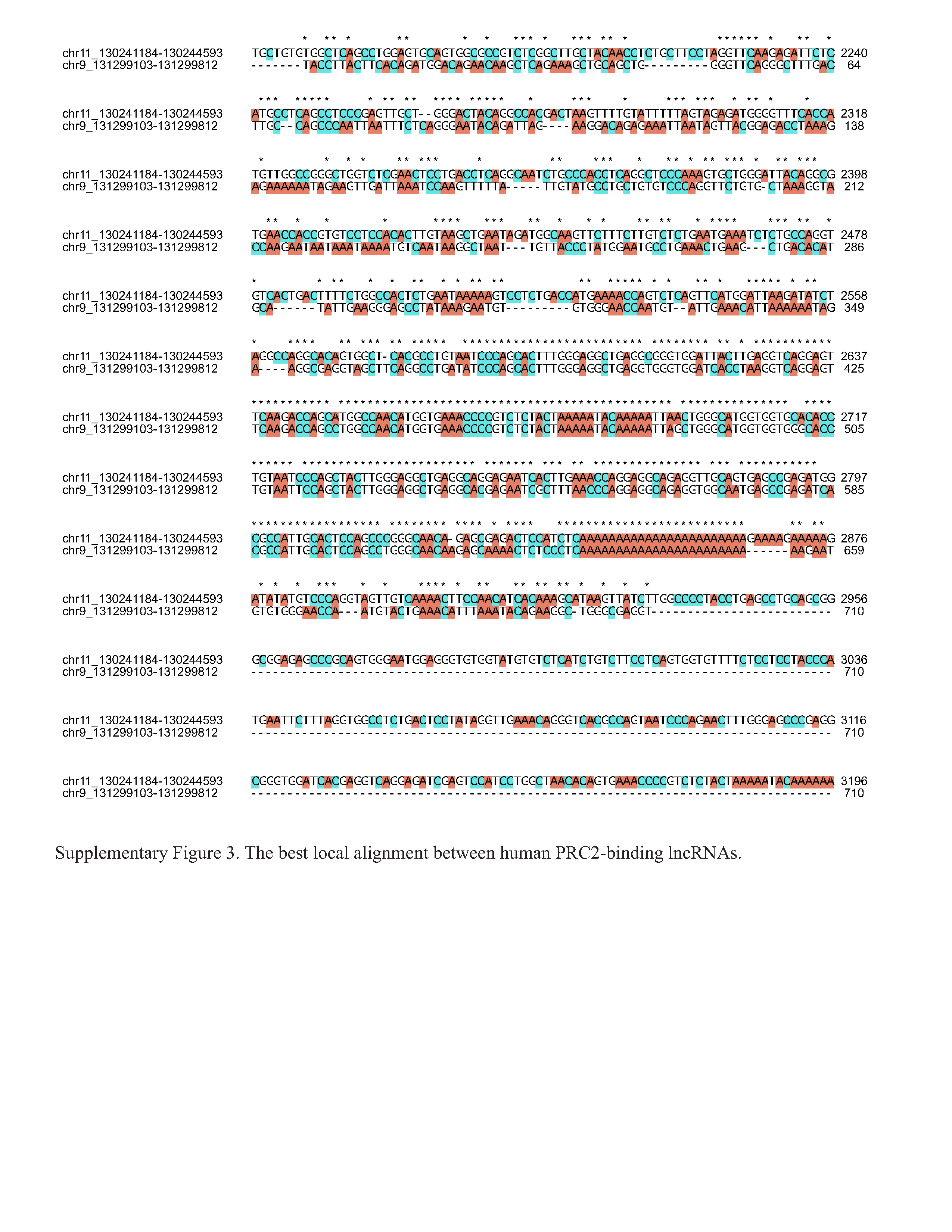

Supplement: Figure S3 — The best local alignment between human PRC2-binding lncRNAs. (TIFF) [file pone.0044878.s004.tiff]

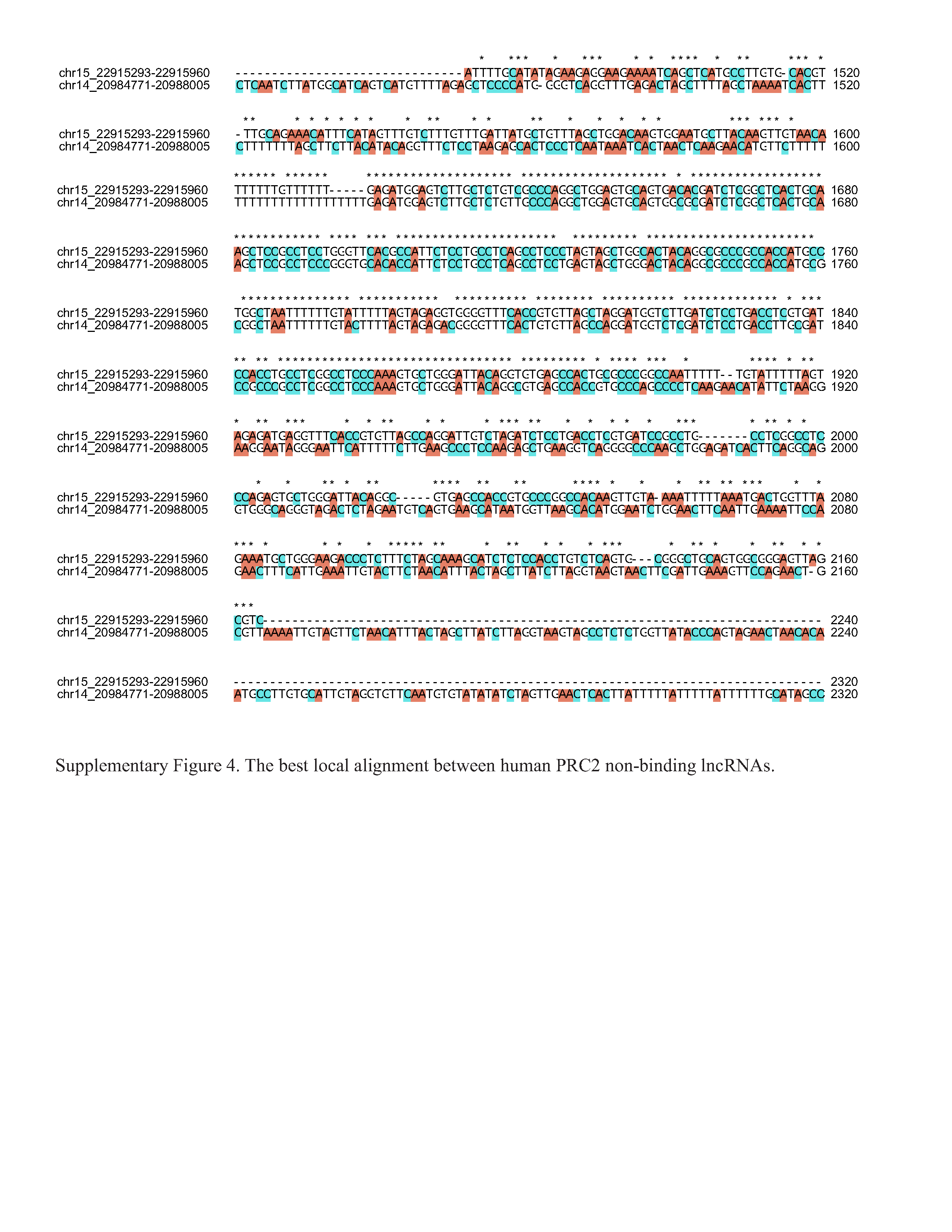

Supplement: Figure S4 — The best local alignment between human PRC2 non-binding lncRNAs. (TIFF) [file pone.0044878.s005.tiff]

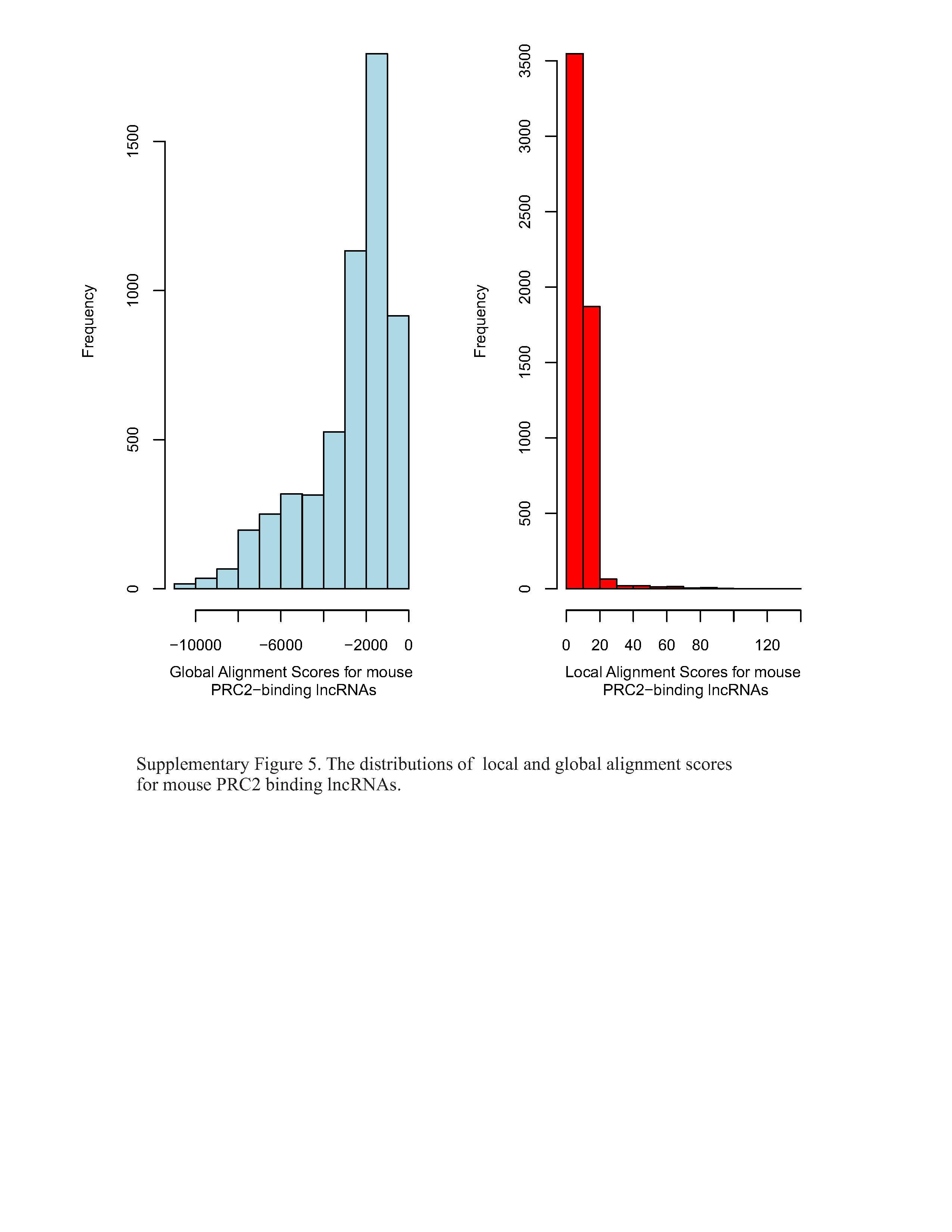

Supplement: Figure S5 — The distributions of local and global alignment scores for mouse PRC2-binding lncRNAs. (TIFF) [file pone.0044878.s006.tiff]

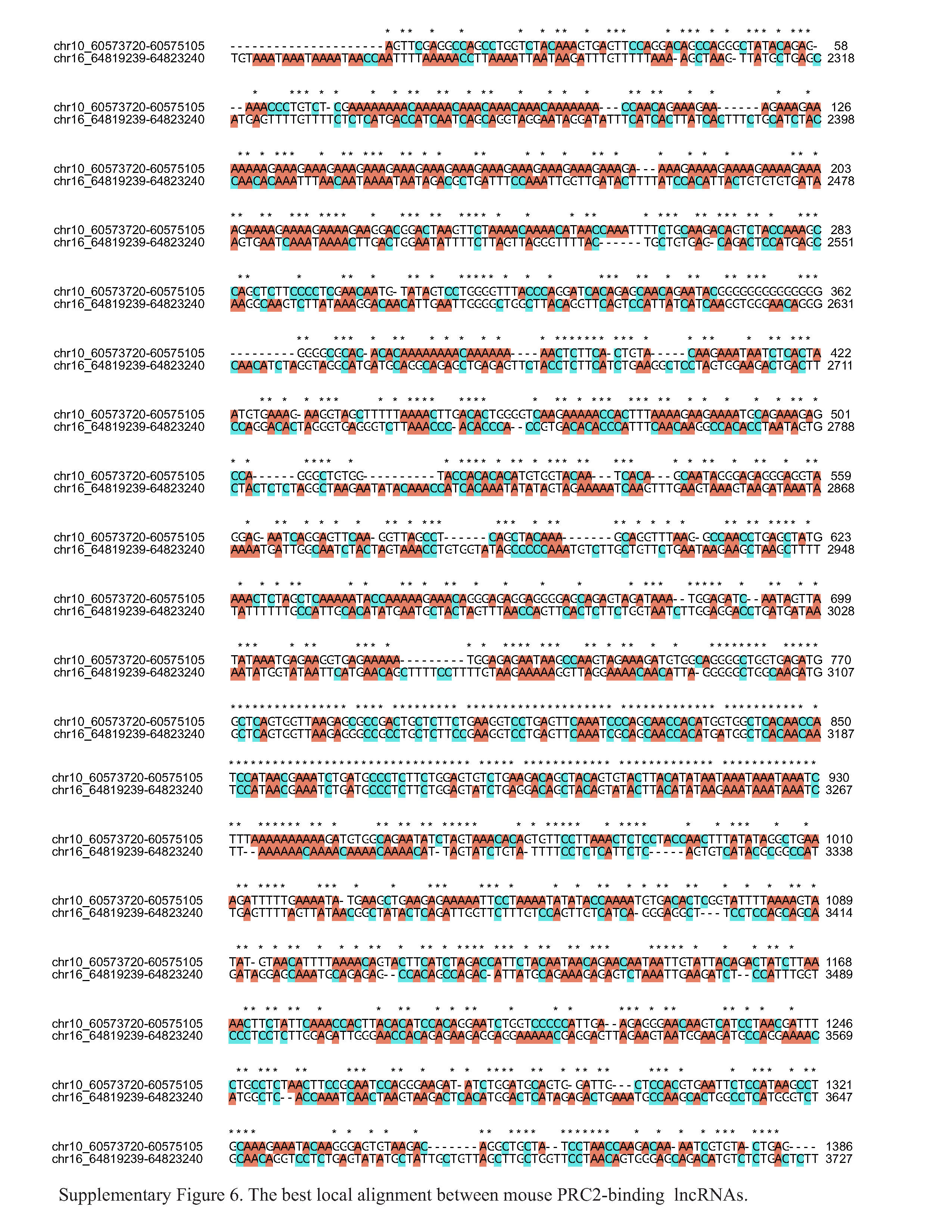

Supplement: Figure S6 — The best local alignment between mouse PRC2-binding lncRNAs. (TIFF) [file pone.0044878.s007.tiff]

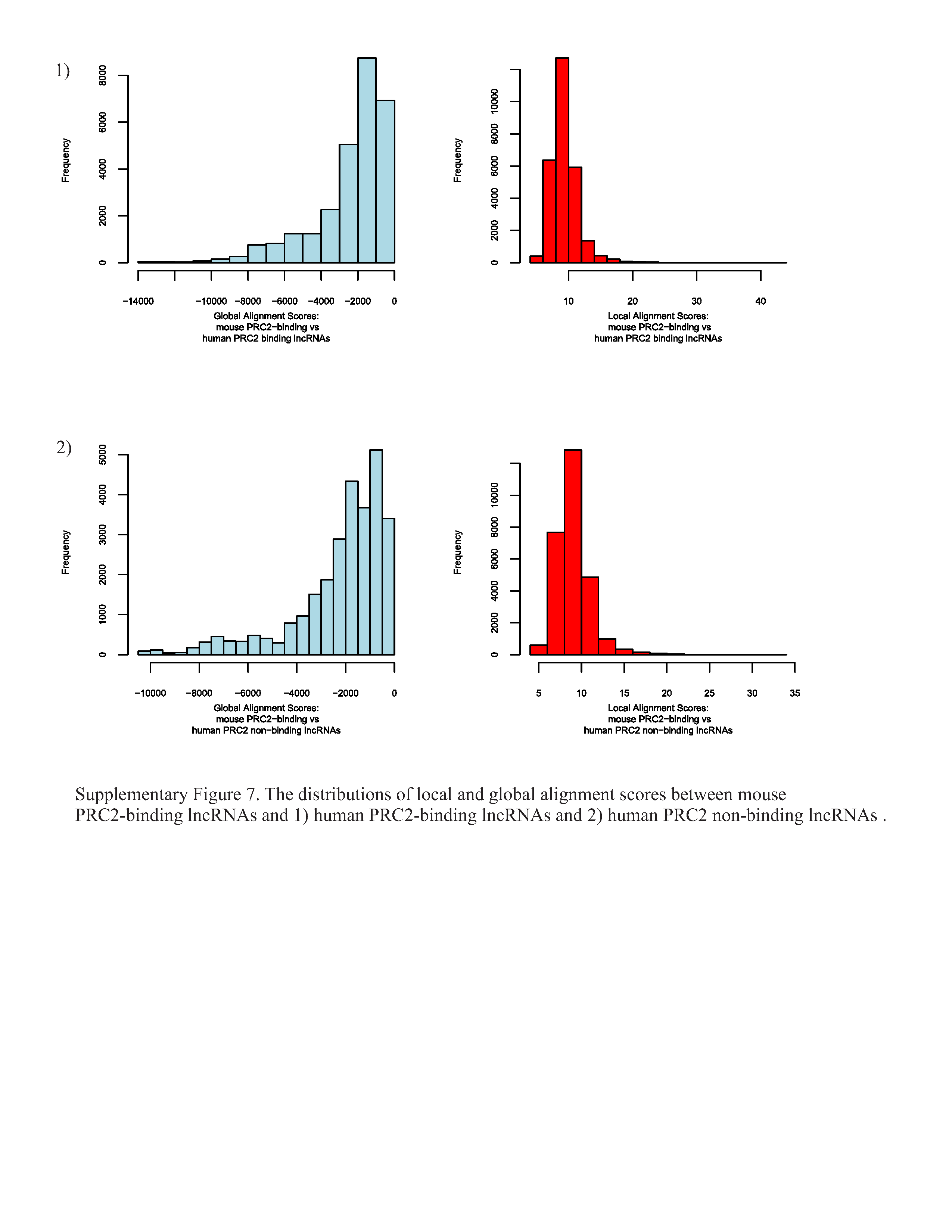

Supplement: Figure S7 — The distributions of local and global alignment scores between mouse PRC2-binding lncRNAs and 1) human PRC2 binding lncRNAs and 2) PRC2 non-binding lncRNAs. (TIFF) [file pone.0044878.s008.tiff]

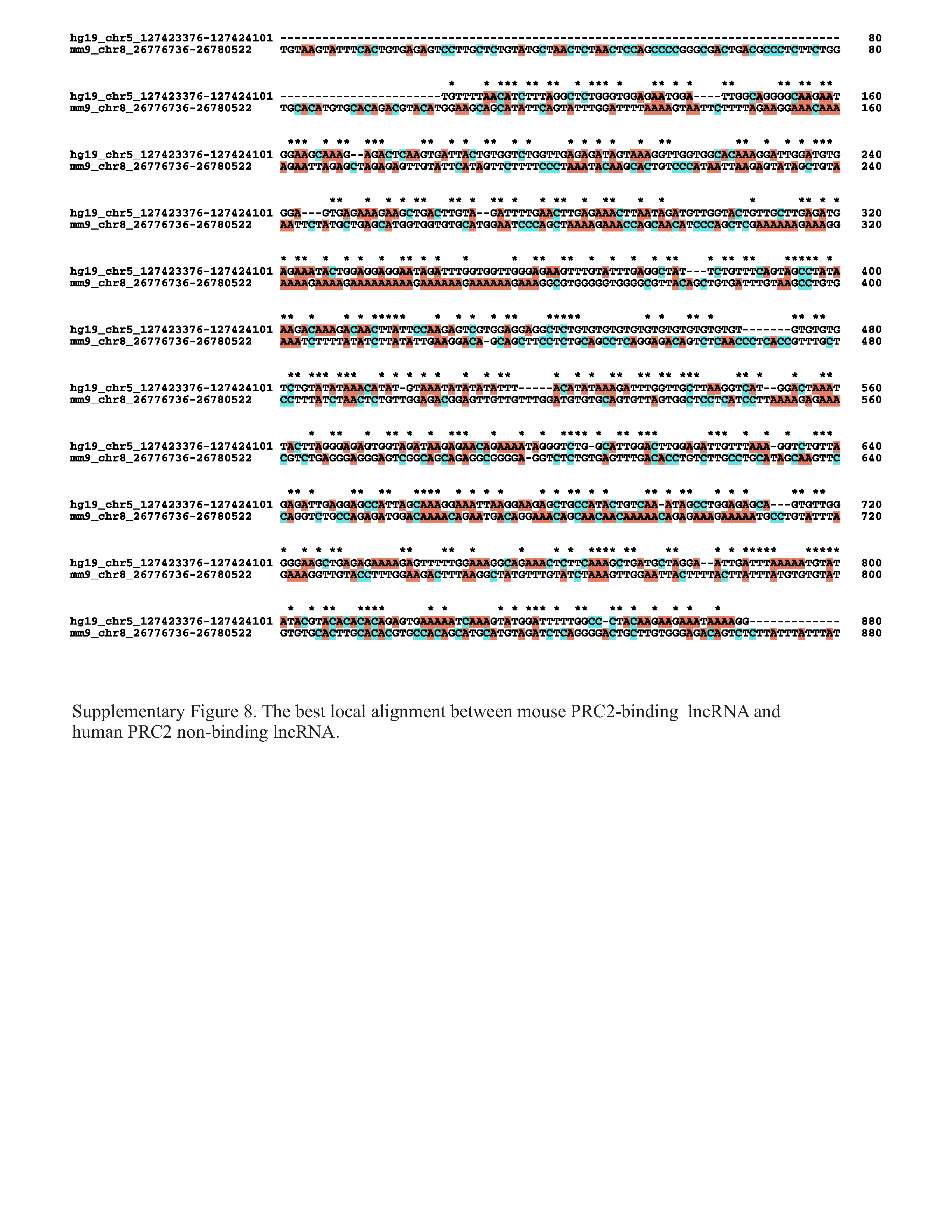

Supplement: Figure S8 — The best local alignment between mouse PRC2-binding and human PRC2 non-binding lncRNAs2. (TIFF) [file pone.0044878.s009.tiff]

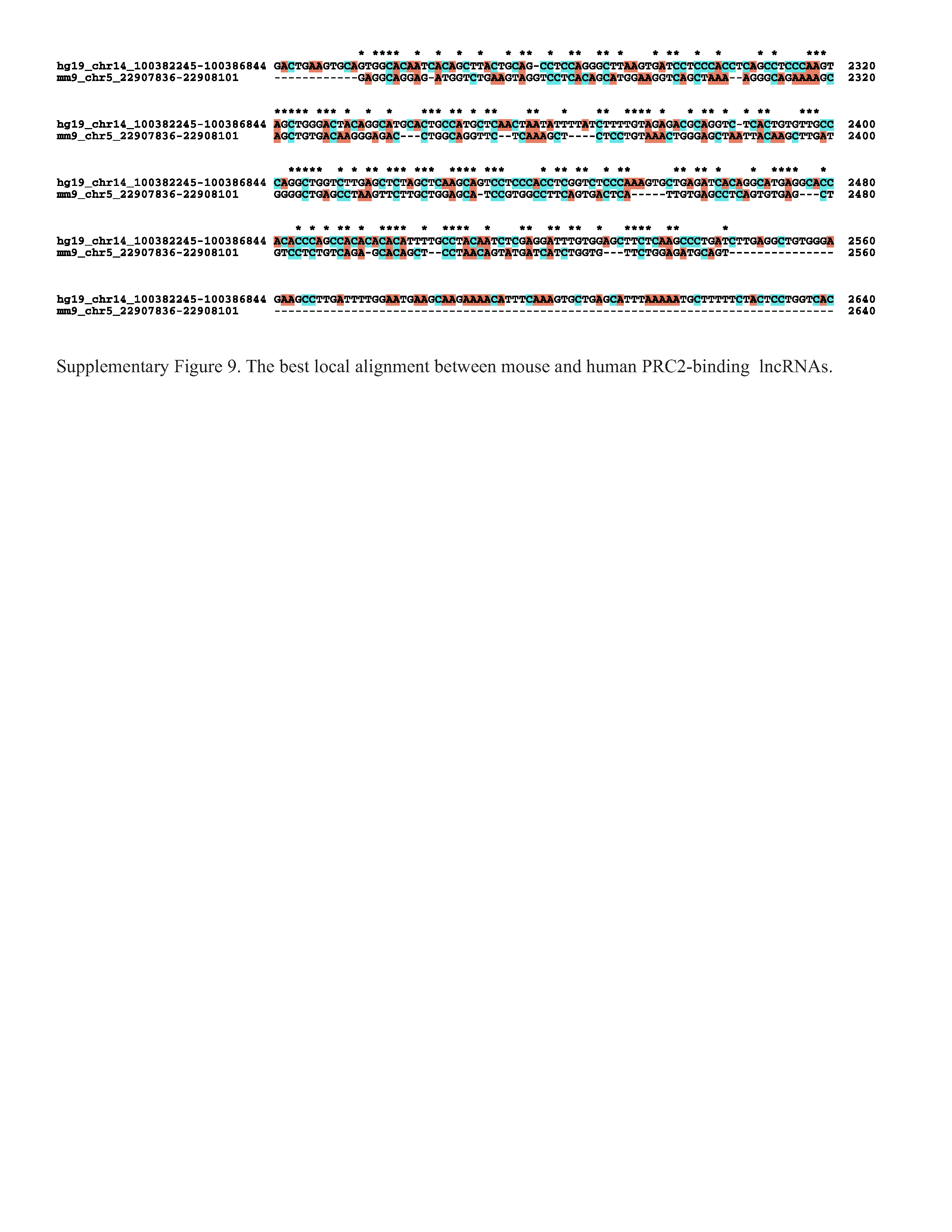

Supplement: Figure S9 — The best local alignment between mouse and human PRC2-binding lncRNAs. (TIFF) [file pone.0044878.s010.tiff]
